# Shear Bond Strength between Orthodontic Brackets and Monolithic 4Y-TZP: An In Vitro Study

**DOI:** 10.3390/ma16145173

**Published:** 2023-07-23

**Authors:** Emre Cakir, Ayse Nurcan Duman, Arzu Zeynep Yildirim, Pinar Cevik

**Affiliations:** 1 Private Practice, Ankara 06420, Türkiye; 2 Department of Prosthodontics, Faculty of Dentistry, Gazi University, Ankara 06490, Türkiye; 3 Department of General Practice and Dental Public Health, School of Dentistry, The University of Texas Health Science Center at Houston, Houston, TX 77054, USA; 4 Houston Center for Biomaterials and Biomimetics, Houston, TX 77054, USA

**Keywords:** bonding, lasers, orthodontic bracket, resin composite, shear strength, yttria stabilized tetragonal zirconia

## Abstract

The aim of this study was to evaluate the effect of different surface treatments on the shear bond strength (SBS) between metal orthodontic brackets and monolithic zirconia surfaces bonded with resin composite. Fifty monolithic zirconia (4Y-TZP) disks were sintered and glazed. Specimens were divided into five groups (*n* = 10) for different surface treatments: control, nano second fiber laser, sandblasting, grinding and tribochemical coating (CoJet Sand 30-μm). Metal orthodontic brackets were bonded to monolithic zirconia surface by two-component orthodontic adhesive. After 500 cycles of thermocycling, shear bond strength values were measured by a universal testing machine at a cross head speed of 0.5 mm/min. The data was recorded as MPa and statistically analyzed with One-way ANOVA, Levene’s LSD tests with Bonferroni corrections. The significance level was α = 0.05. The surface topography of one specimen of each group was evaluated by scanning electron microscopy (SEM). Statistically significant difference was observed among study groups (*p* = 0.018). The lowest shear bond strength was observed in the control group (3.92 ± 1.9). Tribochemical coating showed the highest bond strength (7.44 ± 2.9), which was statistically different from the control and nano second laser (4.3 ± 1.4) groups but not statistically different from grinding (6.15 ± 3.1) or sandblasting (6.47 ± 3.3). SEM images showed comprehensive results of each surface treatment on monolithic zirconia. All failure modes were recorded as adhesive between the composite resin and monolithic zirconia. Based on the findings of this study, it can be concluded that grinding, sandblasting and tribochemical coating techniques showed clinically acceptable bond strength within the range of 6–8 MPa. These surface treatments can be considered suitable for achieving a durable bond between metal orthodontic brackets and monolithic 4Y-TZP ceramic surfaces.

## 1. Introduction

With the increasing demand for adult orthodontic treatment and the growing popularity of esthetic dentistry, clinicians face challenges when bonding orthodontic brackets onto various types of dental restorations compared to enamel [1,2,3]. Zirconium is widely recognized as one of the most used materials for all-ceramic crowns, offering advantages such as esthetic appearance, biocompatibility, and resistance. It is the hardest and strongest ceramic material used in dentistry, displaying mechanical properties comparable to certain metals. The use of zirconia in dentistry has witnessed a surge with the development of CAD-CAM technology. Zirconia exists in three crystallographic structures: monoclinic (from room temperature to 1170 °C), tetragonal (between 1170 °C and 2370 °C) and cubic phase (from 2370 °C up to the melting point of 2680 °C) [4,5,6]. In the transition from the monoclinic phase to the tetragonal phase (when heated above 1170 °C), a volumetric reduction of 5% is observed. During cooling, a growth of 4% occurs. Due to this difference, microcracks occur in pure zirconia. It is a phase change material easily and needs to be stabilized in the tetragonal phase at room temperature. The addition of 3 mol% Y_2_O_3_ to pure zirconium results in the formation of 3Y-TZP (tetragonal zirconium oxide polycrystalline), a fine-grained material commonly used in dentistry. At room temperature, this material primarily consists of a tetragonal phase. The opaque appearance of traditional zirconia (3Y-TZP) does not meet aesthetic expectations, which is why veneering is often recommended. Nevertheless, a common technical issue encountered with veneered zirconia-based restorations is porcelain chipping [4,5]. To address this chipping complication, an alternative approach is to use monolithic zirconia restorations. Recently, the use of monolithically produced zirconia restorations with reduced alumina content has become widespread, particularly in aesthetic areas. This approach eliminates both aesthetic concerns and the risk of chipping or fracturing the outer porcelain veneer. To further enhance aesthetics, partially stabilized zirconia with increased yttrium oxide content has been introduced. This modification introduces a cubic phase in addition to the tetragonal phase. The cubic phase reduces light scattering at the borders of zirconium dioxide crystals, resulting in a more translucent material. These highly translucent zirconia options, such as 4Y-TZP and 5Y-TZP (yttria-stabilized tetragonal zirconium polycrystal), consist of 50% partially stabilized cubic phase. Translucent monolithic zirconia offers a durable prosthetic material option that meets aesthetic expectations, particularly in the anterior region [4,5,6]. However, despite its esthetic advantages, monolithic zirconia poses challenges for orthodontists during adult orthodontic treatment [5].

Achieving adequate bracket bond strength is crucial for successful fixed orthodontic treatment. Maintaining brackets on ceramic restorations, including zirconia, can be problematic, necessitating pre-treatment of ceramic surfaces for successful bonding [7,8]. Various surface treatment methods have been suggested to enhance the bond strength between orthodontic brackets and zirconia surfaces [2,9]. These methods include mechanical modification of the zirconia surface by removing the glaze layer and roughening the surface through sandblasting, grinding or laser etching. However, these methods may lead to irreversible damage or fractures during bracket removal [10,11]. Laser etching, particularly using Er:YAG lasers, has shown promise in roughening the zirconia surface and enhancing micromechanical retention without altering the zirconia structure or causing micro-cracks [3,7,12].

With the introduction of the laser, the idea of using it as a means of reinforcing bond strength has become popular [13,14,15]. Femtosecond lasers are recently used as an alternative for improving the bonding of orthodontic brackets to zirconia surfaces. These lasers generate short pulses, minimizing phase transformations and causing minimal heating effects. They are considered favorable because they do not harm the ceramic surface thermally or mechanically. Additionally, fiber lasers, the latest advancements in laser technology, offer numerous advantages over previous technologies, including improved brilliance, oscillating mode stability, efficiency, packaging possibilities and low maintenance costs [13,14]. While their utilization in dentistry is still limited, fiber lasers have demonstrated potential as alternatives to mechanical surface treatments [14,15]. To date, no studies have evaluated the effect of nano second fiber laser application on monolithic zirconia restorations.

Although several studies have examined the impact of different surface treatment methods on the bond strength of orthodontic attachments to feldspathic and lithium disilicate ceramics, there is limited research regarding the effect of surface treatments on the bond strength of brackets to monolithic zirconia restorations [2,16,17]. An ideal surface treatment for zirconia has not yet been established, one that can withstand intraoral masticatory forces and orthodontic forces during treatment without causing damage during bracket removal. Furthermore, there is insufficient data regarding the success of bonding orthodontic attachments to monolithic zirconia due to its relatively recent introduction in dental practice [18].

Therefore, the objective of this in-vitro study was to evaluate the shear bond strength (SBS) of metal orthodontic brackets to monolithic zirconia subjected to various surface treatments. The null hypothesis tested in this study was that the SBS of orthodontic metal brackets to the surface of monolithic zirconia would not be affected by different surface treatment methods.

## 2. Materials and Methods

A study was conducted using 50 disk-shaped (2 mm thickness, 10 mm diameter) monolithic zirconia specimens (IPS e.max ZirCAD MT BL Multi4, Ivoclar-Vivadent AG, Schaan, Liechtenstein) obtained from pre-sintered zirconia blocks through Computer Aided Design/Computer Aided Manufacturing (CAD/CAM) technology. To achieve a standardized flat surface on the specimens, the specimens were gradually abraded with silicon carbide abrasive papers of varying grain sizes (600, 800 and 1200) under constant water cooling for 20 s. One surface of the specimens was glazed and polished using coarse polishing discs. The polished surfaces were then embedded in acrylic resin molds, and the specimens were randomly divided into 5 groups (*n* = 10) based on different surface treatments:

Group Control: No additional treatment was applied to the polished specimens. Untreated 4 YTZ-P specimens were served as the control group in this study.

**Figure 1:**
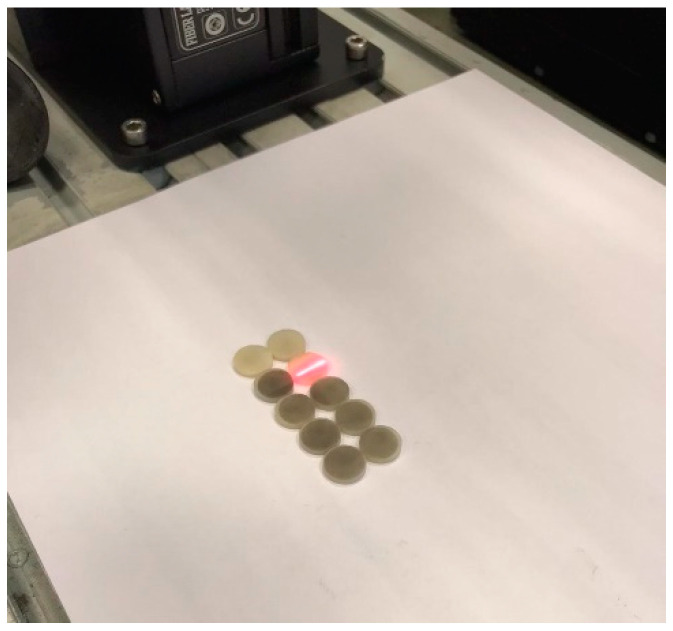
Nano second fiber laser application on monolithic zirconia surface.

Group Laser: Nano second fiber laser irradiation was performed using the following parameters: 2 W power, scanning speed of 500 mm/s, 40 kHz frequency and a spot size of 0.03 × 2. These specific parameters were utilized, according to the manufacturer’s instructions (FiberLAST, FiberLAST A.Ş. Ankara, Turkiye) (Figure 1).

Group Sandblasting: The surfaces were sandblasted with 50 μm Al_2_O_3_ particles using a sandblasting machine under 2.5 bars pressure and at a distance of 10 mm away from the specimen surface. The application time was 10 s for each specimen.

Group Grinding: The surfaces were roughened using ultrafine 20 μm grit cylindrical diamond burrs for 10 s under water cooling. Care was taken during the ultrafine grinding process to prevent exceeding the depth of surface flaws, while focusing solely on the removal of the polished surface.

Group Tribochemical Silica Coating: The surfaces were treated with silica coated Al_2_O_3_ particles (CoJet sand, 3M ESPE, Seefeld, Germany) sprayed onto the zirconia surface for 10 s under 2.5 bar pressure at 10 mm distance.

**Figure 2:**
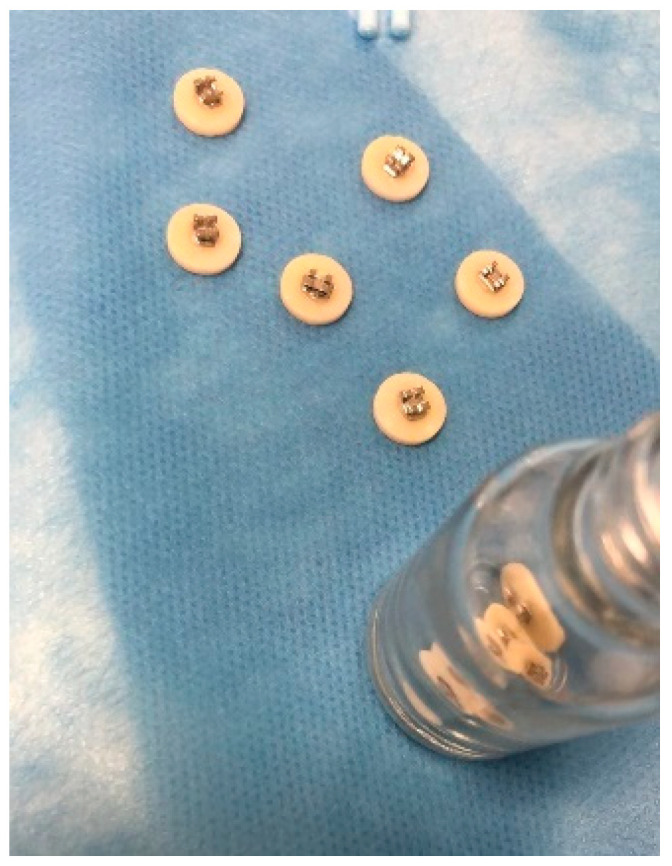
The bonded brackets on the monolithic zirconia specimens.

After the surface treatments, the specimens in each group were cleaned in an ultrasonic cleaner for 15 min and air-dried. BISGMA- and TEGDMA-based primer (3M™ Transbond™ XT Primer, St. Paul, MN, USA) was applied to monolithic zirconia surfaces, according to manufacturer’s instructions. Then, upper incisor metal orthodontic brackets (Leone S.p.A., Florence, Italy) were manually positioned on the zirconia surface, and orthodontic light cure adhesive resin (3M™ Transbond™ XT, St. Paul, MN, USA) was applied to bond the brackets onto the zirconia surfaces. The adhesive was then light cured using a light-emitting diode light-curing unit (LED) for 20 s before testing (Figure 2). All the specimens were subjected to thermocycling 500 times between 5 °C and 50 °C. 

A shear bond test was performed using a Universal testing machine (Lloyd LRX Materials Testing Machine, AMETEK Inc., Berwyn, PN, USA) at a crosshead speed of 0.5 mm/min to measure the strength of the bond between the brackets and the monolithic zirconia surfaces. To evaluate the failure modes of the bond, the surfaces of failed specimens were observed under fluorescent light. The failure modes were categorized as adhesive, cohesive, or mixed. Adhesive failure referred to the separation between the resin and the bracket while cohesive failure indicated failure within the resin or zirconia itself. Mixed-type failure indicated both adhesive and cohesive failure occurring simultaneously in this study.

Statistical analyses were conducted by using the SPSS 23.0 package program (SPSS Inc., Chicago, IL, USA). The significance level for this study was set at *p* < 0.05. Descriptive statistics, including the mean, standard deviation (SD), median, minimum, and maximum SBS (MPa) values, were calculated at 95% confidence intervals. The homogeneity of the data was evaluated using the Levene test. One-way ANOVA and Levene’s LSD multiple comparison tests with Bonferroni corrections were used for post-hoc analysis to determine the statistically significant differences within and between groups (*p* < 0.05).

## 3. Results

**Figure 3:**
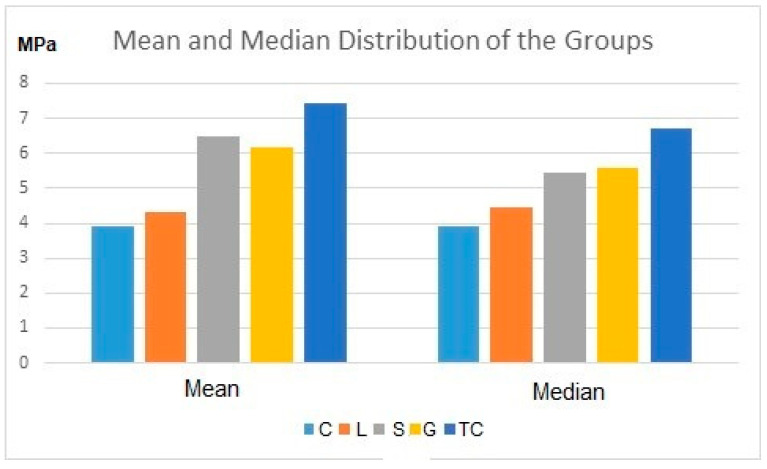
Mean and median values of the SBS value of the study groups in MPa. Note: Control (C), nano second fiber laser (L), Sandblasting (S), grinding (G) and tribochemical coating (TC), respectively.

The descriptive statistics for shear bond strength (SBS) values between metal orthodontic brackets and monolithic zirconia surfaces modified by different surface treatment techniques are presented in Table 1. The table includes mean, standard deviation (SD), median, minimum and maximum SBS values. The mean and median distributions of the groups are shown in Table 1. Additionally, a graph of the groups with means and medians (MPa) can be seen in Figure 3.

The SBS values between metal orthodontic brackets and monolithic zirconia surfaces, after modification with various surface treatment techniques, ranged from (3.92 ± 1.9) to (7.44 ± 2.9) MPa. The tribochemical coating group exhibited the highest SBS value (7.44 ± 2.9) while the control group displayed the lowest SBS value (3.92 ± 1.9). The tribochemical coating group demonstrated a significantly higher SBS value compared to the control (3.92 ± 1.9) and laser (nano second laser) groups (4.3 ± 1.4) (*p* = 0.003 and *p* = 0.010, respectively). The CoJet group did not show a statistically significant difference in SBS value compared to the grinding (6.15 ± 3.1) or sandblasting (6.47 ± 3.3) groups (*p* > 0.05).

Group sandblasting (6.47 ± 3.3) exhibited a statistically higher SBS value compared to the control group (3.92 ± 1.9) (*p* = 0.038). Based on these statistical evaluations, it can be observed that the SBS value of the sandblasting group was superior to the control group, but there was no statistically significant difference between sandblasting (6.47 ± 3.3) and the laser (4.3 ± 1.4), grinding (6.15 ± 3.1) or tribochemical coating groups (7.44 ± 2.9) (*p* > 0.05).

**Figure 4:**
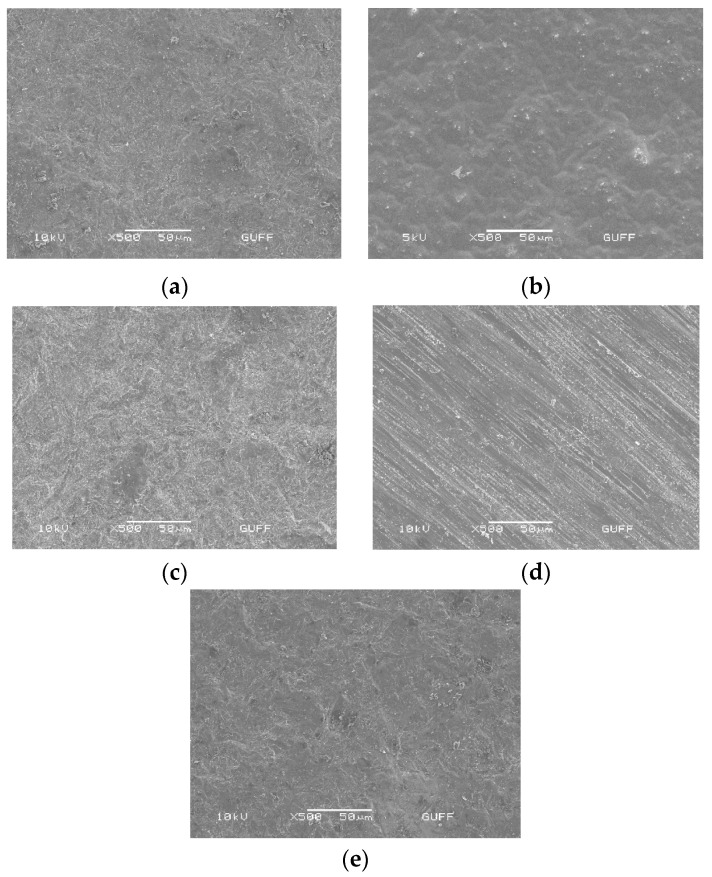
SEM images of the surface treated monolithic zirconia specimens at 500× magnification (**a**) control, (**b**) nano second fiber laser, (**c**) sandblasting, (**d**) grinding, (**e**) tribochemical coating.

SEM images at 500× magnification displayed in Figure 4a–e. SEM images showed comprehensive results of each surface treatment on monolithic Y-TZP specimens. SEM images exhibited all surface treatments, including the control group, affected the surface structure of monolithic zirconia differently from its original condition. The control groups revealed slightly rough surface (Figure 4a). The fiber laser group demonstrated severe melted surface irregularities (Figure 4b). The grinding group exhibited visible scratches, which were parallel to the direction of the diamond burr on the zirconia surface (Figure 4c). The surface of the sandblasting group tended to have slight irregularities consisting of micro porosities (Figure 4d). The surface of the tribochemical coating group of specimens indicated very slight irregularities, which were quite similar to those of the sandblasting group (Figure 4e).

All failure types were recorded as adhesive type in this study.

## 4. Discussion

The results of this in-vitro study showed that SBS between metal brackets and monolithic zirconia surfaces were affected by surface treatments. Therefore, the null hypothesis was rejected in the study.

Currently, there is a growing demand for orthodontic treatment among the adult population. Zirconia, particularly translucent 4Y-TZP ceramics and 5Y-TZP ceramics, known as monolithic restorations, are increasingly used in dentistry due to their improved esthetics and high durability [10,19,20,21]. However, achieving an ideal bond strength with extremely hard and durable monolithic zirconia remains challenging in adult orthodontic treatment [18]. In clinical practice, the bond strength of brackets to ceramic restorations should be sufficiently high to withstand intraoral masticatory forces and orthodontic forces during treatment while also being low enough to prevent permanent damage to the ceramic surface during bracket removal at the end of treatment [7,22].

A minimum bond strength of 5.9–7.8 MPa is clinically required to maintain a good bond between metal brackets and enamel during orthodontic treatment. It has been suggested that a strength of 6–10 MPa is necessary to withstand orthodontic and masticatory forces [23]. It has been proposed that a shear bond strength value close to or higher than 6 MPa is suitable for the clinical use of brackets [24]. Thurmond et al. reported that bond strengths exceeding 13 MPa resulted in cohesive fractures on the ceramic surface [25]. Adhesive failure indicates that the bond strength between the composite and the metal bracket is stronger than the bond strength between the composite and the ceramic [26]. In this study, none of the surface treatments resulted in bond strengths exceeding 7 MPa, even after 500 cycles of thermocycling. Furthermore, all recorded failure types were classified as adhesive, suggesting the absence of significant ceramic damage. 

To enhance the bond strength between the orthodontic bracket and Y-TZP ceramic, surface treatment methods are required because the glazed surface of ceramic materials is inert [7,24,27]. These methods can be mechanical, chemical or a combination of both. It is widely recognized in the literature that surface roughening is necessary to achieve sufficient bond strength on zirconia restorations. Mechanical methods such as grinding or sandblasting can help create micromechanical retentions. The grain size of the burr used in grinding, as well as the application pressure, time and speed, can influence the surface roughness [20,28].

In this study, the grinding, sandblasting and tribochemical coating groups demonstrated higher bond strength within the acceptable threshold range (6–8 MPa) compared to the control group (*p* < 0.05). It should be noted that since the restorations typically remain in the mouth after debonding the brackets, it is important to avoid excessive roughening of the surfaces during pretreatment or debonding. After debonding, the ceramic surface should be polished using a porcelain polishing kit to avoid damage to the ceramic. Therefore, ultrafine diamond burrs were utilized in the grinding group to prevent crack initiation within the ceramic. Scanning electron microscope (SEM) images of the grinding group showed scratches on the monolithic zirconia surface, but no cohesive fractures were observed in the grinding groups after undergoing 500 cycles of thermocycling. However, it is important for clinicians to exercise caution and avoid deep roughening during grinding, opting for ultrafine grinding instead. In this study, the zirconia surfaces were roughened using ultrafine cylindrical diamond burrs at 40,000 rpm for 10 s with high-speed rotary instruments under water cooling to remove the polished layer on the zirconium surface. Adequate shear bond strength (6.15 ± 3.1 MPa) was achieved with the grinding method.

Sandblasting and tribochemical silica coating are effective and simple methods to create micromechanical retention on the zirconia surface [29,30,31]. These methods increase the surface area, roughness and wettability of the zirconia, which in turn enhances the bond strength [18,32,33]. It is important to note that, while sandblasting can increase the strength of Y-TZP, it has the potential to compromise the compressive stress layer and to promote crack propagation [12]. Excessive air pressure during sandblasting can also cause undesirable phase inversion from tetragonal to monoclinic zirconia [34]. Therefore, not only the size and pressure of the sand but also factors such as application distance, time and angle of the device can affect the effectiveness of the sandblasting process [18].

In this study, sandblasting at a pressure of 2.5 bars resulted in a significant increase in shear bond strength compared to the control group, which is consistent with previous reports [8,35,36]. Furthermore, sandblasting with Al_2_O_3_ exhibited higher bond strength than grinding although there was no statistical difference between them. This finding is in line with several previous studies [8,16,18,29,32,37,38,39]. The improved bonding values observed with sandblasting in this study may be attributed to the adhesion of the adhesive cement into the minor porosities created by the sandblasting process, leading to micromechanical locking. Importantly, no cracks or fractures were observed on the zirconia surfaces subjected to sandblasting with Al_2_O_3_ in this study, indicating the safety and effectiveness of this method. 

The use of silica particles in surface treatment methods serves the purpose of roughening the ceramic surface mechanically. However, in addition to the mechanical effect, silica particles also have a chemical effect. In the tribochemical coating technique, namely CoJet system, silanated Al_2_O_3_ sand particles modified with silica are employed for sandblasting [12,19,40]. These modified particles, with a grain size of 30 μm, induce two significant processes when blasted onto ceramic surfaces. Firstly, micromechanical retention occurs as the particles become embedded in the zirconia surface, leading to improved adhesion. Secondly, a chemical reaction takes place between the embedded silica and alumina particles and a silane coupling agent [19,31,33]. This chemical reaction further enhances the bond between the coating and the ceramic surface.

It is important to note that, while silica is effective for glass-contained ceramics, different surface treatments, such as the use of phosphate monomers, may be required for Y-TZP surfaces (yttrium-stabilized tetragonal zirconia polycrystalline). The specific type of ceramic material being used may necessitate different surface treatment approaches.

The study findings indicated that the scanning electron microscopy (SEM) images of the tribochemical coating group showed similar differences compared to the sandblasting group. This suggests that both methods have comparable effects on the ceramic surface, supporting their effectiveness in achieving micromechanical retention and improving bonding.

The researchers proposed that the high bond strength observed in the tribochemical coating group can be attributed to both mechanical locking and chemical retention. Besides the physical interlocking of the coating with the ceramic surface, the chemical reaction between the embedded silica and alumina particles and the phosphate monomer of the resin cement played a significant role in achieving strong bonding. It is likely that the adhesive cement used in the study flowed into the micro-porosities created by the surface treatment, and the presence of the phosphate monomer further enhanced the bond strength. Consequently, the tribochemical coating group exhibited the highest bond strength, with an average value of 7.44 ± 2.9, specifically in monolithic zirconia.

These findings suggest that the tribochemical coating, which provides both mechanical and chemical retention, along with the use of a phosphate monomer in combination with the adhesive cement, can significantly improve bond strength when bonding resin cement to monolithic zirconia surfaces. These results align with previous studies [1,12,40] that also demonstrated increased bond strength on monolithic zirconia surfaces with tribochemical coating surface treatment. Based on these findings, it can be inferred that both tribochemical coating and sandblasting can be utilized to achieve durable bond strength between orthodontic brackets and resin cement. It is also important to note that sandblasting as a surface treatment technique requires careful consideration due to potential risks. It has the possibility of irritating intraoral tissues and causing mechanical damage to soft tissues. Additionally, there is a risk of inhalation of aluminum oxide particles [22]. However, these risks can be minimized in clinical practice by using effective aspiration devices to minimize the dispersion of particles. In our study, we took precautions to minimize the potential damages by conducting the sandblasting and silica coating processes in short time intervals and from a fixed distance, while also isolating the working environment to prevent dust dispersion. Similarly, when selecting a surface treatment technique, it is advisable to use rubber dam covers in the mouth to minimize potentially hazardous effects on the lungs and gingiva. These precautions aim to ensure the safety of patients and clinicians during the surface-conditioning process [22].

The principal effect of laser energy is the conversion of light energy into heat, and the most important interaction between laser and substrate is the absorption of the laser energy by the substrate [12]. During conventional laser surface treatment, steep local temperature changes in the heating and cooling phases generate internal tensions and microcracks, which can weaken the material and tooth [41,42]. It is necessary to use appropriate laser operating parameters. It has been stated that there is no thermal effect occurrence within the specimen during material processing with nano second ultrafast fiber laser, which eliminates the formation of cracks, because highly focused laser energy causes evaporation before the melting phase of the material [41]. However, fiber lasers are primarily used in the industrial sector for material processing, and the use of fiber lasers in dental material processing has been limited [41]. 

Recent studies have reported an increase in bond strength between titanium surfaces and resin cement or acrylic structures when treated with nano-second fiber laser application [14,41]. Fiber laser has been suggested as a potential alternative to traditional sandblasting methods. It should be noted that, in comparison to the sandblasting group, the laser application resulted in decreased bond strength in this study, which could be attributed to variations in the surface properties of titanium and monolithic zirconia structures [14,41]. SEM images of the laser-treated specimens revealed the presence of melted areas on the monolithic surface, indicating the generation of high surface energy during laser application. There are few studies evaluating the effect of fiber lasers on the bond strength of metal brackets to different ceramic materials. Fornaini [13] demonstrated that the utilization of 1070 nm pulsed fiber lasers for the surface treatment of lithium disilicate ceramics is effective and damage-free. Figure 1 in this study shows that the laser application had diverse effects on the surface of the monolithic zirconia specimens, resulting in a noticeable black coloring effect. These findings highlight the importance of considering the potential color alteration on light white/natural surfaces when employing fiber laser treatments. Additionally, the nano second fiber laser group exhibited a lower shear bond strength value (4.3 ± 1.4) in this study, which was below the acceptable range for orthodontic bonding. In future research, it is recommended to examine the effect of nano second fiber laser with power levels below 2 W and above 1 W on the SBS between brackets and monolithic surfaces. This investigation would help determine whether applying lower laser energy can result in improved performance for the bonding between metal brackets and monolithic 4 YTZ-P zirconia surfaces. Additionally, it would be beneficial to assess the physical effects on the monolithic zirconia surface when using lower energy power in nano second fiber lasers. These additional experiments would provide valuable insights into optimizing the laser treatment parameters and enhancing the bonding strength between metal brackets and monolithic zirconia surfaces.

Within the limitations of this study, generalization of results to the clinical setting should be done with caution. The forces applied to the brackets under experimental conditions are different from the forces that occur in vivo. In the mouth, brackets are influenced by different types of stretching, shear, rotational and combined forces, and the forces that are applied for the removal of brackets in the clinic are different from the pure shear force that is applied gradually in a laboratory. It should also be noted that achieving optimal bond strength is influenced by various factors beyond just the ceramic surface treatment method. These factors include the type of ceramic material, the type of bracket and its base surface design or retention mode, properties of the bonding adhesive, the light curing source used, the oral environment and the experience of the clinician [2,7,20,28,43]. It was suggested that optimizing the polishing process can eliminate the monoclinic phase, preventing phase transformation and crack propagation in monolithic zirconia [43,44]. It can be recommended to perform a polishing procedure on the monolithic zirconia surface after debonding to prevent any adverse effects on the material. This step can help restore the surface finish and maintain the integrity of the material. Additionally, in future studies, it would be beneficial to investigate X-ray diffraction analysis on the surface treated zirconia specimens. This analysis can provide insights into the relative percentage of the monoclinic phase in the differently treated zirconia specimens. Such investigations would contribute to a better understanding of the structural changes induced by various surface treatments and their impact on the overall performance of the zirconia material.

In conclusion, following appropriate surface roughening techniques can lead to an acceptable surface structure and successful bonding during surface treatment in patients with monolithic zirconia restorations.

## 5. Conclusions

Based on the mean SBS values and SEM images, several conclusions can be drawn from this in vitro study:

1. The shear bond strength (SBS) values between metal orthodontic brackets and monolithic zirconia, modified by different surface treatment methods, ranged from (3.92 ± 1.9) to (7.44 ± 2.9).
2. The tribochemical coating group exhibited the highest bond strength (7.44 ± 2.9), which was not statistically different from the grinding (6.15 ± 3.1) or sandblasting (6.47 ± 3.3) groups.
3. The bond strength achieved using the nano second fiber laser (4.3 ± 1.4) fell below the acceptable threshold range of 6–8 MPa.
4. Additionally, the nano second fiber laser significantly altered the color of the monolithic zirconia specimens.
5. The grinding, sandblasting and tribochemical coating techniques demonstrated clinically acceptable bond strength within the range of 6–8 MPa, suggesting their potential suitability for use in clinical practice.

## Figures and Tables

**Table 1:** SBS results of the groups with mean (MPa), standard deviation (SD), median, minimum, and maximum values (*p* < 0.05).

	Control	Nano Second Fiber Laser	Sandblasting	Grinding	Tribochemical Coating
N	10	10	10	10	10
Mean	3.927 ^a^	4.328 ^a^	6.477 ^ac^	6.155 ^ac^	7.448 ^c^
SD	1.900	1.466	3.317	3.125	2.978
Median	3.929	4.448	5.450	5.599	6.700
Minimum	0.423	2.297	3.578	3.078	3.518
Maximum	6.641	6.434	15.662	15.177	13.789

Note. Different superscript letters indicate statistical difference between groups in the same row (*p* < 0.05).

## Data Availability

Data is contained in Supplementary Materials.

## Supplementary Materials

The following supporting information can be downloaded at: https://www.mdpi.com/article/10.3390/ma16145173/s1, Table S1: Samples’ information data.
